# Physical activity during pregnancy: Essential steps for maternal and fetal health

**DOI:** 10.1177/1753495X221122540

**Published:** 2022-08-24

**Authors:** Margie H Davenport, Melanie Hayman

**Affiliations:** 3158 University of Alberta, Canada Email: mdavenpo@ualberta.ca; 6939 Central Queensland University, Australia

Over the last 50 years, our understanding of the impact of physical activity on maternal and fetal health has profoundly changed. Prior to the first guidelines put forth by the American College of Obstetricians and Gynecologists in 1985, pregnant individuals were advised to rest and relax due to concerns about overexerting the ‘fragile’ female physique. But over the last half century, antenatal physical activity has emerged as a powerful preventative tool to reduce major pregnancy complications. Depression, pre-eclampsia and gestational diabetes are reduced by 40–67% without increasing the risk of adverse pregnancy outcomes, including miscarriage, preterm delivery or small for gestational age baby.^1,2^ Recent position statements and a review article published in the *New England Journal of Medicine* now advocate for moderate-intensity physical activity as a safe and accessible frontline treatment to prevent the development of preeclampsia.^3^ This is a major shift in our view of physical activity as activity restriction was previously advised for those at high risk.

Global guidelines recommend accumulating at least 150 min of moderate-intensity physical activity (e.g. brisk walking or other activities where your heart rate goes up and you can talk but not sing) over 3 or more days of the week to derive clinically meaningful benefits.^4–7^ However, it is important to screen for medical reasons where moderate-to-vigorous activity may not be recommended. The recently developed Get Active Questionnaire for Pregnancy (https://csep.ca/2021/05/27/get-active-questionnaire-for-pregnancy/) has been internationally recognized and endorsed as a tool that can be self-completed by pregnant individuals. This tool identifies the minority of individuals at risk for contraindication(s) to physical activity where additional screening is warranted.^8^ Yet even those identified as having an absolute contraindication to physical activity are encouraged to engage in activities of daily living, as a complete cessation of activity (bed rest) is not endorsed by obstetric organizations due to established potential for harm, with no evidence of benefit.^9^

Although patients and practitioners may envision that individuals need to engage in breathless, sweaty activities, the reality is the majority of evidence is based on studies of antenatal walking. Much less is actually known about high-intensity, or long-duration activity during pregnancy. Thus, those who wish to engage in activities that substantially exceed current recommendations (e.g. athletes), are encouraged to do so in consultation with their obstetric care provider. However, the limited empirical evidence supporting these activities needs to be balanced with the potential adverse impact on maternal mental and physical health, caused by reducing activities to the point of ‘detraining’ in previously highly active individuals. Encouraging, albeit limited literature, synthesized from pregnant elite athletes that continue to train and compete during pregnancy, demonstrate similar pregnancy outcomes to less active individuals.^10^

There are a number of safety considerations for physical activity during pregnancy; while empirical evidence supporting these is limited, the theoretical concerns are sufficient and warrant discussion. Pregnant individuals are recommended to avoid activities with a high risk of ‘bumping the bump’ such as horseback riding, downhill skiing, non-stationary biking and other activities. Although there is a level of protection to the fetus through the uterus and amniotic fluid, direct trauma to the abdomen can cause harm to the fetus through loss of amniotic fluid, placental abruption and/or premature labour. Other activities such as scuba diving and high altitude exercise (for those living at sea level) are not recommended due to potential harm to the fetus.^4,11^ There is also limited evidence regarding the safety of pregnant women exercising in excessive heat (especially with high humidity such as hot yoga), due to an elevated risk of dehydration and hyperthermia. Instead, exercise is recommended to be performed under cooler conditions such as in air conditioning or by avoiding the midday heat by exercising in the shade or in the early morning.

All pregnant individuals and their obstetric care providers need to be aware of signs and symptoms (recognized as ‘red flags’) during exercise where activity should cease immediately and medical advice sought. These include the onset of persistent excessive shortness of breath, severe chest pain, regular and painful uterine contractions, vaginal bleeding, persistent loss of fluid from the vagina and persistent dizziness or faintness that does not resolve upon rest. These signs and symptoms can represent the initiation of labour or be a symptom of a clinically relevant complication.

Pregnancy can be a physically and mentally challenging time. There will be days individuals will feel tired or unwell; encouraging patients to adapt or reduce their activity as needed is important. The key to better maternal and fetal health is to focus on long-term engagement in physical activity during pregnancy – even engaging in activity levels well below current recommendations has substantial health benefits. Our previous work demonstrates that by engaging in at least 10 min each day of moderate-intensity walking, the odds of developing preeclampsia are reduced by 25%.^2^ Further, when bumping up moderate-intensity walking to 15 min per day, we found additional benefits including a 25% reduction in the odds of developing gestational hypertension or excessive gestational weight gain.^2,12^

Exercise is safe, accessible and beneficial for the vast majority of pregnant individuals. Yet, the prescription of physical activity and exercise to reduce the risk of complications in the obstetric population is highly under-utilized.^13^ Research highlights that obstetric care providers lack appropriate support (training and resources) to initiate conversations about antenatal physical activity, and often provide advice that is overly conservative. Having been extensively involved in developing evidence-based guidelines,^4,7^ we understand that exercise prescription can be difficult to fit into a busy clinical practice. If you only have a minute, relay to your patients that accumulating 150 min of moderate-intensity activity (e.g. brisk walking) each week is associated with at least a 25% reduction in the risk of major pregnancy complications without increasing the risk of miscarriage, preterm birth or small baby. Obstetricians and other health professionals are encouraged to utilize the Get Active Questionnaire for Pregnancy to help guide their conversations with pregnant individuals. Now is the time to change our conversation with patients from what are the risks associated with physical activity to what are the potential harms of *not* engaging in activity.
